# Treatment Option for Abernethy Malformation—Two Cases Report and Review of the Literature

**DOI:** 10.3389/fped.2020.497447

**Published:** 2020-10-27

**Authors:** Yuese Lin, Xuandi Li, Shujuan Li, Hongjun Ba, Huishen Wang, Ling Zhu

**Affiliations:** ^1^Department of Paediatric Cardiology, Heart Center, The First Affiliated Hospital, Sun Yat-sen University, Guangzhou, China; ^2^Key Laboratory on Assisted Circulation, Ministry of Health, Guangzhou, China

**Keywords:** abernethy malformation, hepatopulmonary syndrome, congenital portosystemic shunts(CPSS), pulmonary arteriovenous fistula (pavf), transcatheter shunt closure

## Abstract

**Background:** Abernethy malformation is a rare vascular anomaly of the portal venous system, which is also known as congenital portosystemic shunts (CPSS). The clinical manifestations of this anomaly can be serious, including hepatopulmonary syndrome(HPS), which can lead to significant hypoxemia and cyanosis.

**Case Presentation:** This study reports two cases of patients with Abernethy Malformation. Case 1 was a 6-year-old boy whose blood oxygen saturation was 78%. Case 2 was a 6-year-old girl who had a history of open heart surgery and residual cardiac left to right shunt, whose blood oxygen saturation was 83%. These two children had unexplained cyanosis and were diagnosed with pulmonary arteriovenous fistula by contrast echocardiography with agitated saline. A selective retrograde catheter angiography confirmed the presence of a portosystemic shunt. Case 1 was a type I Abernethy malformation and did not receive any specific treatment and could only wait for liver transplantation. Case 2 was with type II Abernethy and underwent transcatheter closure of the CPSS. A 20mm-diameter, 14mm-long Vascular Plug (SHSMA Inc, Shanghai, China) was used to occlude the shunt.

**Results:** In case 1, the boy developed deteriorating cyanosis and dyspnea on exertion. In case 2, the exercise tolerance of the patient improved after shunt closure. During a follow-up of 3 years, her blood oxygen saturation increased from 83 to 98%.

**Conclusion:** The results indicate that children with unexplained cyanosis require special attention since these patients may have Abernethy malformation, and part of them could be treated by transcatheter occlusion with a good outcome. The key to treatment is how it is diagnosed and carefully assessed.

## Background

Abernethy malformation, also known as congenital portosystemic shunts (CPSS), is a rare vascular anomaly of the portal venous system. Clinical manifestations can be serious, including hepatopulmonarysyndrome (HPS), which can lead to significant hypoxemia and cyanosis. The management of this anomaly depends on the type of malformation. There have been few reports and there is little literature about treatment choices and outcomes for children with Abernethy malformation.

This study discusses two cases of patients with Abernethy Malformation. Case 1 was a 6-year-old boy with type I Abernethy malformation, who did not receive any specific treatment and could only wait for liver transplantation. The boy developed deteriorating cyanosis and dyspnea on exertion at follow-up. Case 2 was a 6-year-old girl with type II Abernethy who underwent transcatheter closure of the CPSS. Her exercise tolerance improved after shunt closure and resting oxygen saturation returned to normal.

## Case Presentation

### Case 1

**Figure 1:**
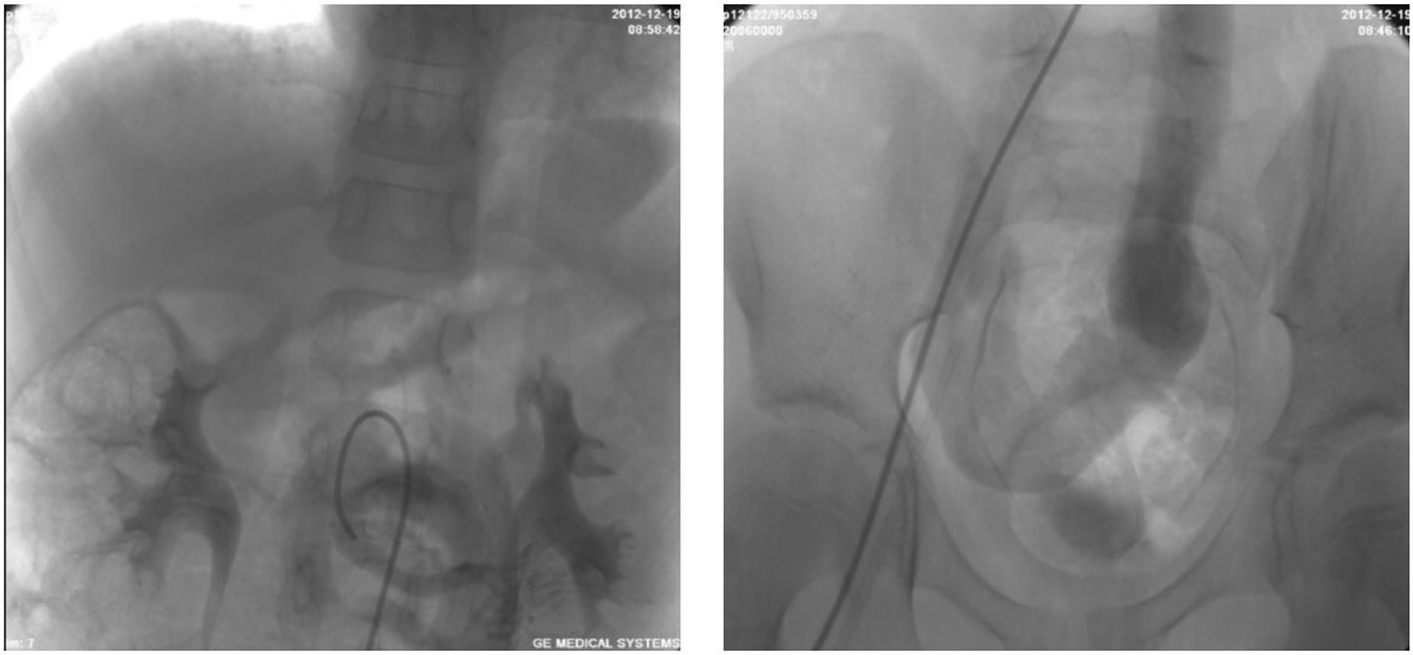
Superior mesenteric arteriography demonstrating an end-to-side porto-caval shunt with no intrahepatic portal vein.

**Figure 2:**
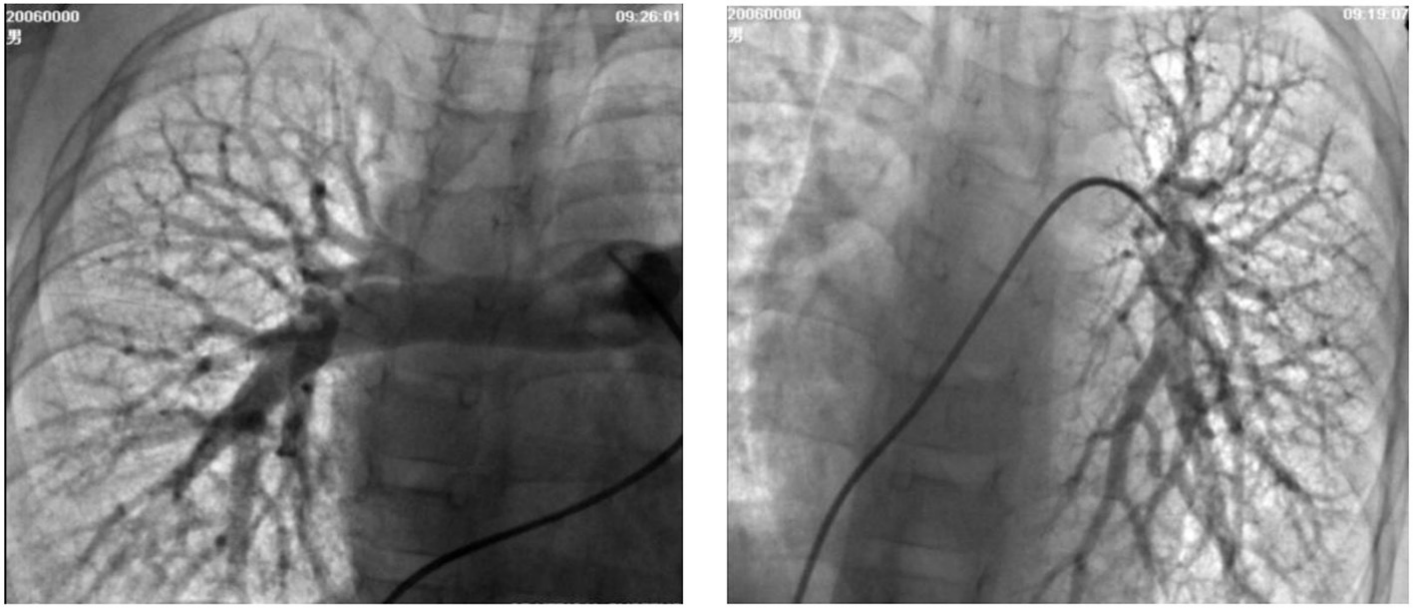
Selective lung angiography showing diffuse small reticular end-on vessel pattern on both lungs and immediate imaging of the left atrium, suggestive of pulmonary arteriovenous fistula (PAVF).

A 6-year-old boy was evaluated for a 2-year history of unexplained cyanosis and dyspnea on exertion. Physical examination on admission revealed cyanosis and digital clubbing with a resting oxygen saturation of 78% on room air, mild hepatomegaly, and jaundice were also noted. Lung sounds were clear and cardiac examination showed normal heart sounds without any murmurs. Blood test results showed a hemoglobin level of 160 g/L and hematocrit of 48%. Liver function test results demonstrated mild jaundice with a total bilirubin of 41 μmol/L. Serum ammonia was elevated to 49 μmol/L (normal range 9–33 μmol/L) but no evidence of hepatic encephalopathy was found. The coagulation profile was slightly abnormal with a prothrombin time of 16.7 s, an international normalized ratio (INR) of 1.43, and activated partial thromboplastin time (APTT) of 45 s. His chest X-ray showed increased pulmonary vascular patterns. ECG was unremarkable and the echocardiography examination revealed a mild left-sided volume overload (a left ventricular end-diastolic dimension of 43 mm). A contrast echocardiography with agitated saline showed the appearance of microbubbles in the left atrium after four cardiac cycles, which suggested pulmonary arteriovenous fistula. A contrast-enhanced computed tomography (CT) scan of the abdomen revealed no sign of portal vein and demonstrated an abnormal communication between the splanchnic vein and systemic venous circulation in which intestinal and splenic venous blood bypassed the liver completely and drained directly into the inferior vena cava (IVC). Cardiac catheterization was performed for further examination of the unexplained cyanosis. A superior mesenteric arteriography, obtained in the venous phase, confirmed an end-to-side porto-caval shunt with no intrahepatic portal vein, superior mesenteric vein joined the splenic vein and then the confertus splanchnic vein bypassed the liver and drained into the IVC (Figure 1). Right cardiac catheterization showed a normal pulmonary artery pressure of 15 mmHg and selective lung angiography showed diffuse small reticular end-on vessel pattern on both lungs and immediate imaging of the left atrium, suggestive of pulmonary arteriovenous fistula (PAVF) (Figure 2). Based on these findings, the patient was diagnosed with hepatopulmonary syndrome secondary to type I Abernethy malformation.

### Case 2

**Figure 3:**
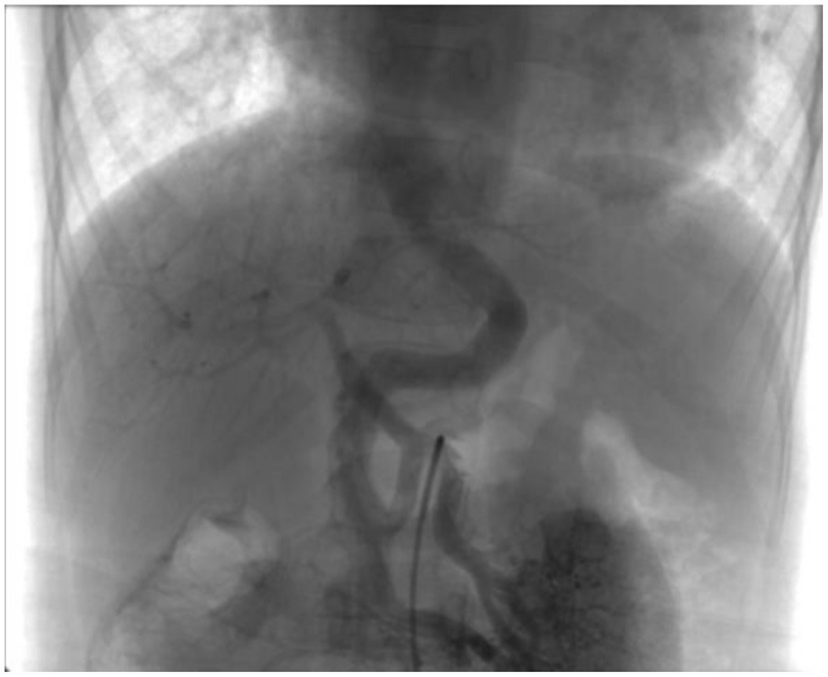
Selective retrograde catheter angiography demonstrating the presence of a side-to-side shunt between the portal vein and IVC along with an acceptable intrahepatic portal venous system.

A 6-year-old girl was admitted to our department with a 3-year history of unexplained cyanosis, decreased exercise tolerance, and slow growth [weight of 14.5 kg (72% of expected)]. She had a history of a ventricular septal defect and patent ductus arteriosus and underwent open heart surgery when she was 1-year-old. A physical examination revealed that the girl was hypoxic with a resting oxygen saturation of 83% on room air, hepatomegaly, and digital clubbing were also found. No evidence of hepatic encephalopathy was found. Cardiac examination demonstrated a grade 3–4/6 systolic ejection murmur in the second and third left intercostal spaces radiating to the back. Blood test results showed a hemoglobin level of 150 g/L and hematocrit of 44%. The liver function test and coagulation tests results were normal. A chest X-ray showed mild cardiomegaly and increased pulmonary vascular markings. An echocardiography showed a 5 mm residual left to right shunt between the left ventricle and right atrium and mild pulmonary valve stenosis. A contrast echocardiography with agitated saline showed the appearance of microbubbles in the left atrium after four cardiac cycles, suggestive of pulmonary arteriovenous fistula. Cardiac catheterization was performed for further examination. Selective retrograde catheter angiography confirmed the presence of a side-to-side shunt between the portal vein and IVC along with an acceptable intrahepatic portal venous system (Figure 3). Right cardiac catheterization showed a mean pulmonary pressure of 25mmHg. Given these findings, the patient was diagnosed with hepatopulmonary syndrome secondary to type II Abernethy malformation.

## Interventions and Results

Because Case 1, the boy with type I Abernethy malformation, did not receive any specific treatment and could only wait for liver transplantation. He developed deteriorating cyanosis and dyspnea on exertion during the following 6 years. Resting oxygen saturation on room air decreased from 78 to 74%. The hemoglobin levels remained the same but total bilirubin was elevated from 41 to 52 μmol/L and serum ammonia was elevated from 49 to 56 μmol/L. No evidence of hepatic encephalopathy was found. An echocardiography revealed worsening left sided volume overload and the left ventricular end-diastolic dimension increased from 43 to 52 mm.

**Figure 4:**
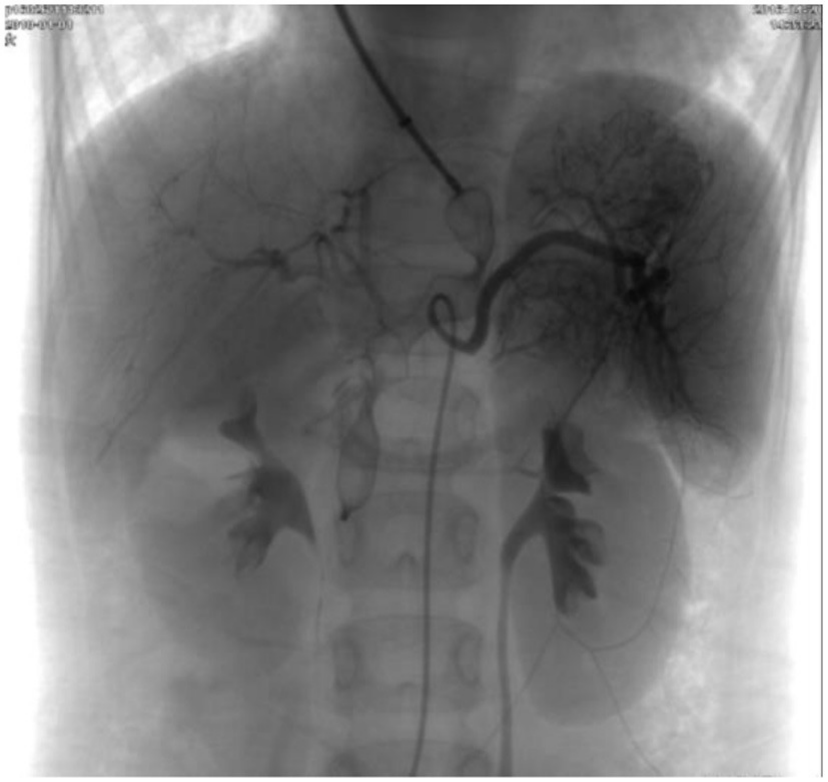
Repeated superior mesenteric arteriography showing complete occlusion of the shunt and a good hepatic portal circulation.

In case 2, the girl with type II Abernethy malformation underwent transcatheter closure. She received percutaneous device closure of the shunt under general anesthesia through the left subclavian vein approach. The left subclavian vein and right femoral artery were percutaneously accessed. The shunt luminal measured 8 mm in diameter and the portal vein pressure mean gradient was 7 mmHg before the procedure. A 20 mm-diameter, 14 mm-long Vascular Plug (SHSMA Inc, Shanghai, China) was used to occlude the shunt deployed through a 7 French long sheath. Repeated pressure measurement of the portal vein after the occlusion appeared to 10 mmHg, suggested the patency of the portal system. After deployment, repeated superior mesenteric arteriography showed complete occlusion of the shunt and a good hepatic portal circulation (Figure 4). At a follow-up of 3 years, her exercise tolerance improved after shunt closure, and her hemoglobin level returned to normal, and resting oxygen saturation increased from 83 to 98% on room air. During follow-up, a contrast-enhanced CT scan of the abdomen showed a normal hepatic portal circulation and there was no obstruction of the device to the hepatic veins or renal veins. No complication occurred during a 4-year follow-up after the shunt occlusion.

## Discussion

The common cause of central cyanosis in children includes various types of heart and lung diseases, hemoglobinopathy, and diffuse pulmonary arteriovenous fistula, etc, which may lead to elevated concentration of reduced hemoglobin in the blood that results in cyanosis.

Etiology of the diffuse pulmonary arteriovenous fistula may be the result of congenital haemagiectasis of the lung or it could be secondary to certain diseases such as Abernethy malformation, which can lead to hepatopulmonary syndrome(HPS), causing significant hypoxemia and cyanosis. Hepatopulmonary syndrome is due to vasodilation and angiogenesis in the pulmonary vascular bed, which results in ventilation-perfusion imbalance, limitation of oxygen diffusion, and arteriovenous shunts (AVS) (1).

Abernethy malformation is a rare vascular anomaly of the portal venous system, which is also known as congenital extrahepatic portosystemic shunts (CPSS) (2). It was first described by Abernethy in 1793 (3). Morgan and Superina classified it into 2 types according to how abnormal communication takes place between the portal vein and systemic venous circulation (4). In type I Abernethy malformation, an end-to-side porto-caval shunt exists with no intrahepatic portal vein, the portal vein bypasses the liver and drains into the IVC directly. In type II Abernethy malformation, a side-to-side porto-caval shunt exists and intrahepatic portal venous is preserved but hypoplastic. Abernethy malformation may lead to severe complications such as liver tumors, hepatic encephalopathy, hepatopulmonary syndrome, or pulmonary hypertension. The treatment option was based on the pattern of the shunt (5). Due to the absence of intrahepatic portal vein, liver transplantation is considered to be the only effective therapeutic approach for symptomatic type I Abernethy malformation, since no medical treatment proved effective. Surgical ligation or transcatheter closure of the shunt is the therapeutic option for type II Abernethy malformation (6, 7).

The two cases we report were both patients with Abernethy Malformation who presented with unexplained and progressive cyanosis. Case 1 was with type I Abernethy malformation, and they did not receive any specific treatment and could only wait for liver transplantation. Case 2 was with type II Abernethy malformation. We performed percutaneous device closure of the shunt for this patient according to catheter angiography and have a good outcome. To the best of our knowledge, the successful transcatheter closure of congenital extrahepatic portosystemic shunts has been reported in around 10 children with type II Abernethy malformation including our case from this report (8–12). Exercise tolerance and resting oxygen saturation of these patients increased after the procedure.

The results indicate that special attention should be paid to children with unexplained cyanosis. Children with unexplained cyanosis especially for those born acyanotic and who develop unexplained and progressive cyanosis postnatally. These cases should undergo a series of examinations including blood gas analysis, chest X-ray checks, echocardiography, and microbubble tests with contrast echocardiography, and CT-scans, etc. Abdominal ultrasound only allows for the measurement of the liver, assessment of the development, and vasculature of the portal vein, which is of little significance in the diagnosis of CPSS. An enhanced abdominal CT scan is required in cyanotic children with pulmonary arteriovenous fistula (PAVF). Further cardiac catheterization like radiography and angiography tests should be carried out of the congenital extrahepatic portosystemic shunt is suspicious, and treatment options depend on the specific type of Abernethy malformation. Whether the patient had ever undergone examination and treatment (including heart surgery), the diagnoses of Abernethy malformation should not be missed.

### Clinical Significance

Early diagnosis and appropriate management of Abernethy malformation may lead to improved prognosis. Our study aims to point out the clinical significance of the comprehensive evaluation for patients with unexplained cyanosis where there is a suspicion of pulmonary arteriovenous fistula and hepatopulmonary syndrome. Symptoms like exertional dyspnea, cyanosis, pulmonary hypertension are only partially improved in some patients with delayed treatment. Early diagnosis, careful evaluation, and appropriate management may result in fully relieving symptoms including cyanosis and improved quality of life. Besides routine echocardiography and abdominal color doppler ultrasound examination, a thorough assessment including contrast echocardiography and especially enhanced abdominal CT scan are extremely meaningful and particularly important in determining the etiology of hepatopulmonary syndrome.

### Limitations

Abernethy malformation is rare and this case report only outlines preliminary experiences with transcatheter treatment for type II Abernethy malformation. Long-term follow-up will focus on prognosis and any adverse complications after shunt closure is required and necessary. As the number of clinical cases and accumulation of experience increases in the future, we will continue to summarize and improve technology.

## Conclusion

Since some patients may have Abernethy malformation and part of them could be treated by transcatheter occlusion with a good outcome, it is important to make an early diagnosis and carefully assess and avoid missed or misdiagnosis. Early intervention treatment may reduce complications and improve survival rates.

In summary, transcatheter closure of the abnormal extrahepatic portosystemic shunt is an effective and safe treatment for symptomatic type II Abernethy malformation. Early diagnosis and appropriate management may lead to improved prognosis and continued close and long-term follow-up after shunt closure is required.

## Data Availability Statement

All datasets generated for this study are included in the article/Supplementary Material.

## Ethics Statement

The studies involving human participants were reviewed and approved by the First Affiliated Hospital of Sun Yat-sen University, Guangzhou, China. Written informed consent to participate in this study was provided by the participants' legal guardian/next of kin. Written informed consent was obtained from the minor(s)' legal guardian/next of kin for the publication of any potentially identifiable images or data included in this article.

## Author Contributions

All authors listed have made a substantial, direct and intellectual contribution to the work, and approved it for publication.

## Conflict of Interest

The authors declare that the research was conducted in the absence of any commercial or financial relationships that could be construed as a potential conflict of interest.
